# Cocaine Administration and Its Withdrawal Enhance the Expression of Genes Encoding Histone-Modifying Enzymes and Histone Acetylation in the Rat Prefrontal Cortex

**DOI:** 10.1007/s12640-017-9728-7

**Published:** 2017-04-10

**Authors:** Anna Sadakierska-Chudy, Małgorzata Frankowska, Joanna Jastrzębska, Karolina Wydra, Joanna Miszkiel, Marek Sanak, Małgorzata Filip

**Affiliations:** 1 Institute of Pharmacology Polish Academy of Sciences, Department of Pharmacology, Laboratory of Drug Addiction Pharmacology, Polish Academy of Sciences, ul. Smetna 12, 31-343 Krakow, Poland; 2 Laboratory of Molecular Biology and Clinical Genetics, Medical College, Jagiellonian University, ul. Skawinska 8, 31-066 Krakow, Poland

**Keywords:** Cocaine self-administration, Gene expression, Histone-modifying enzymes, Microarray, Posttranslational histone modifications

## Abstract

Chronic exposure to cocaine, craving, and relapse are attributed to long-lasting changes in gene expression arising through epigenetic and transcriptional mechanisms. Although several brain regions are involved in these processes, the prefrontal cortex seems to play a crucial role not only in motivation and decision-making but also in extinction and seeking behavior. In this study, we applied cocaine self-administration and extinction training procedures in rats with a yoked triad to determine differentially expressed genes in prefrontal cortex. Microarray analysis showed significant upregulation of several genes encoding histone modification enzymes during early extinction training. Subsequent real-time PCR testing of these genes following cocaine self-administration or early (third day) and late (tenth day) extinction revealed elevated levels of their transcripts. Interestingly, we found the enrichment of *Brd1* messenger RNA in rats self-administering cocaine that lasted until extinction training during cocaine withdrawal with concomitant increased acetylation of H3K9 and H4K8. However, despite elevated levels of methyl- and demethyltransferase-encoded transcripts, no changes in global di- and tri-methylation of histone H3 at lysine 4, 9, 27, and 79 were observed. Surprisingly, at the end of extinction training (10 days of cocaine withdrawal), most of the analyzed genes in the rats actively and passively administering cocaine returned to the control level. Together, the alterations identified in the rat prefrontal cortex may suggest enhanced chromatin remodeling and transcriptional activity induced by early cocaine abstinence; however, to know whether they are beneficial or not for the extinction of drug-seeking behavior, further in vivo evaluation is required.

**Electronic supplementary material:**

The online version of this article (doi:10.1007/s12640-017-9728-7) contains supplementary material, which is available to authorized users.

## Introduction

Substance use disorder (SUD) is a chronic, relapsing brain disease characterized by compulsive drug taking and seeking. Due to its complex nature, understanding of the cellular and molecular mechanisms of SUD is still incomplete. SUD is often described as maladaptive neuronal plasticity leading to long-term behavioral abnormalities and gene expression alterations throughout the mesolimbic dopamine system (Walker et al. 2015). For example, repeated intake of cocaine promotes changes in the patterns of gene expression that underly addiction as well as neuroadaptations (Nestler 2004; Kalivas 2005). Increasing evidence suggests that cocaine may induce both transient and persistent changes in gene expression that are important in subsequent addicted phenotype (Walker et al. 2015; Yuferov et al. 2005). The persistence of cocaine craving and risk of relapse have been attributed to drug-induced epigenetic mechanisms that seem to be attractive candidates explaining long-lasting drug-induced molecular alterations; however, it is still undiscovered whether drug-induced changes in chromatin structure remain persistent. To date, the majority of studies on epigenetic mechanisms involved in cocaine drug-taking and drug-seeking behaviors have focused on histone posttranslational modifications (PTMs) in the nucleus accumbens (NAc) as a major region of integration for the rewarding system. In fact, drugs of abuse such as cocaine alter the expression levels of DNA- and histone-modifying enzymes in the NAc (Renthal et al. 2007; LaPlant et al. 2010; Maze et al. 2010). However, a study by Wang et al. (2010) did not show changes in the global acetylation level of H3K9/14ac and H4K5/8/12ac during cocaine self-administration in the rat NAc, while the first evidence provided by Kumar et al. (2005) indicated that cocaine has the potential to induce changes in histone modifications at the gene promoters in the rat striatum. Moreover, a subsequent study demonstrated that the histone methyltransferase G9a was persistently downregulated in mouse NAc following repeated cocaine treatment (Maze et al. 2010). In addition, acute and chronic exposure to psychostimulants increases global or site-specific levels of acetylated histone H3 or H4 in the striatum and NAc (Kumar et al. 2005; Shen et al. 2008; Rogge and Wood 2013).

Currently, many studies have focused on the molecular effects of cocaine self-administration; however, changes during drug abstinence (with extinction training) could also be very important for further relapse prevention therapy. Several lines of evidence suggest that extinction is the gradual reduction of a conditioned response when the conditioned stimulus (cue) is no longer paired with the unconditioned stimulus (drug) and forms a “new” and “active” learning that not only deletes original drug-memory but instead competes with initial memory for control of behavior (Gass and Chandler 2013). The formation of a new inhibitory “extinction memory” utilizes several key brain regions for the expression of extinction drug-seeking behavior including prefrontal cortex (PFC) and hippocampus. Importantly, the PFC is associated not only with working memory, impulsivity, motivation, and decision-making but also with extinction behavior (Gass and Chandler 2013), so it seems reasonable to assume that epigenetic changes may occur in this brain structure.

In our study, we performed microarray analysis in the PFC in early (3 day) cocaine abstinence with extinction training and, ultimately, selected nine genes for further real-time PCR testing in rats undergoing cocaine self-administration as well as early and late (10 day) time points of extinction training. The selected set of genes included genes encoding histone-modifying enzymes and histone proteins that may control the chromatin state. Additionally, we determined the levels of acetylation and methylation marks on specific lysine residues of histones H3 and H4 during early cocaine abstinence to keep track of whether changes in gene expression influenced histone modifications. Taken together, our results revealed upregulation of several genes engaged in chromatin remodeling and the enrichment of lysine acetylation mainly during early cocaine abstinence.

## Materials and Methods

### Subjects

Male Wistar rats (290–350 g; Charles River Laboratories, Germany) were used for the study, which was carried out in accordance with the European Directive 2010/63/EU and with approval from the Local Ethics Commission in the Institute of Pharmacology, PAS. Animals were housed in standard home cages (five rats per cage) in a temperature-controlled room with a 12-h light-dark cycle (lights on at 6:00 a.m.) with ad libidum access to food (Labofeed pellets) and water (except for a day with initial lever press training and a day with retraining following the surgery where water was provided for 2 h/day after 2-h water training sessions). After surgery for catheter implantation, animals were housed individually; however, visual, olfactory, and acoustic contacts remained possible.

### Cocaine Self-Administration and Extinction Training

The surgical and cocaine self-administration procedures used were described previously (Sadakierska-Chudy et al. 2016). Briefly, animals were trained initially for water reinforcement for 1 week with increasing fixed ratio (FR) requirements (FR1, FR3, and finally, FR5). Rats were then implanted with indwelling jugular catheters and allowed 7 days for recovery. The catheters were flushed daily with 0.2 ml of an antibiotic solution of cefazolin (10 mg/kg; Tarfazolin, Polfa, Poland) dissolved in heparinized saline (70 U/ml in 0.9% sterile saline: Polfa, Poland) or with heparinized saline. After recovery, the rats were randomly assigned to either cocaine self-administration or a yoked group (cocaine or saline). All self-administration experiments were conducted in standard operant chambers (Med Associates, St. Albans, USA) equipped with two levers (active and inactive), a cue light, a home light, and a tone generator (2000 Hz). For rats self-administering cocaine, presses on the active lever (FR5) resulted in a single 0.1 ml infusion of cocaine (0.5 mg/kg/infusion; cocaine HCl (Sigma-Aldrich, USA) in sterile 0.9% NaCl) and a 5-s light and tone cue. A 20-s time-out period followed each infusion (inactive lever presses had no consequence). Rats underwent 2-h daily sessions 6 days/week for a minimum 12 days. The criterion for acquisition was a 3-day period during maintenance in which the number of active lever presses varied by 10% or less. The experimental events were scheduled, and data collection was controlled via computer with Med Associates interface and software (Med-PC IV software, MED Associates Inc., Vermont, USA). The extinction training sessions occurred in the same operant chambers and lasted for 2 h daily; however, presses on the previously active lever no longer produced drug or presentation of the drug-paired cues.

To distinguish the pharmacological effects from motivation, the yoked procedure was used. Subsets of animals serving as yoked cocaine and yoked saline controls received cocaine or saline infusion each time that their active cocaine counterpart received a cocaine infusion.

### Experimental Groups

**Fig. 1:**
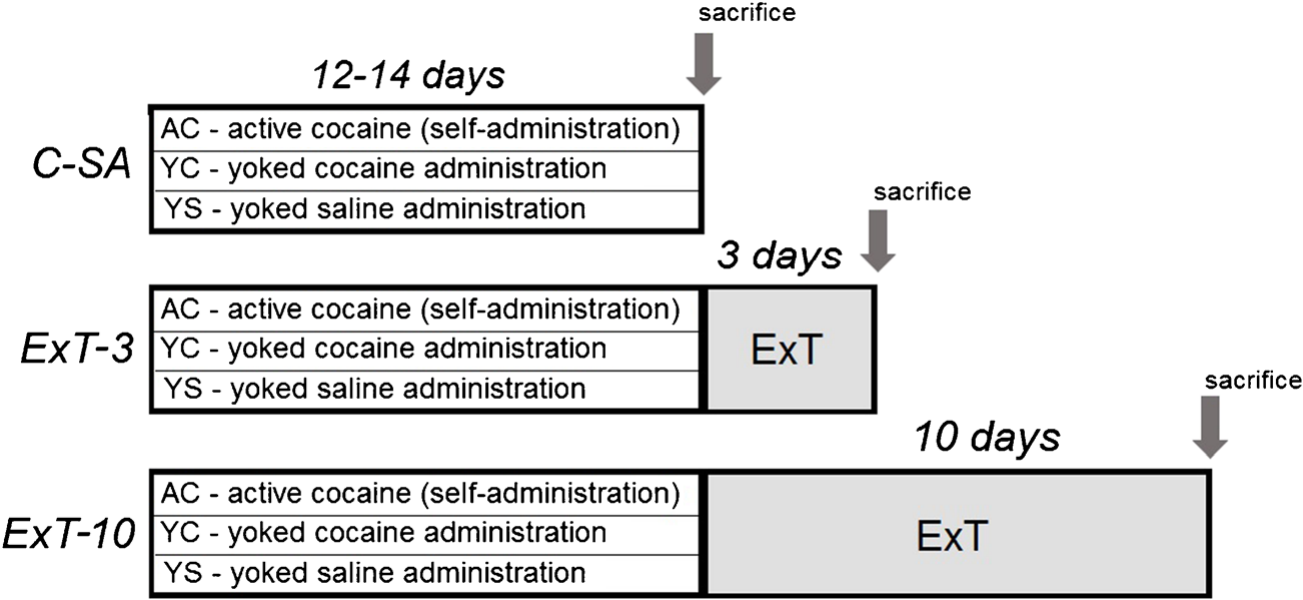
Diagram illustrating the experimental procedure and resulting groups. *C-SA* cocaine self-administration experiment; *ExT-3* third day of cocaine abstinence with extinction training; *ExT-10* tenth day of cocaine abstinence with extinction training. Animal groups: *AC* active cocaine rats, *YC* yoked cocaine rats, *YS* yoked saline rats (control)

The experiment consisted of 12–14 days of cocaine self-administration (*C-SA*) and early (3 days) and late (10 days) extinction from cocaine self-administration (*ExT-3* and *ExT-10*, respectively). In each experiment, three groups of rats were tested: 1—active cocaine (AC), 2—yoked cocaine (YC), and 3—yoked saline (YS) (Fig. 1).

### Tissue Collection and RNA Extraction

The animals were sacrificed immediately following the last 2-h experimental session. The dissected PFC was rapidly placed on dry ice and frozen at −80 °C for further analyses.

Isolation of RNA was performed using the RNA/DNA/PROTEIN Purification Plus Kit (Norgen Biotek, Canada) following the manufacturer’s protocol*.* Briefly, the frozen brain structures (10–15 mg) were homogenized using the Bioprep-24 Homogenizer (Aosheng, China) (30 s at 3000 rpm, then 2 × 30 s at 2500 rpm) in the presence of ceramic beads (*ø* = 2.8 mm) and 350 μl of lysis buffer. RNA samples were eluted in nuclease-free water preheated to 60 °C, followed by removal of traces of DNA by treatment with DNase I (Qiagen, USA) using the RNA Clean-Up kit (Syngen, Poland) according to the manufacturer’s recommendation, after which the purified RNA was eluted in nuclease-free water and stored at −80 °C until further analysis.

The quantity and quality of the extracted RNA were determined using a NanoDrop ND-1000 Spectrophotometer (Thermo Scientific, USA) and agarose gel electrophoresis. Additionally, RNA integrity was evaluated using chip-based capillary electrophoresis with an RNA 6000 Nano Chip Kit and an Agilent Bioanalyzer (Agilent Technologies, USA).

### Microarray Analysis

The Rat 4x44K Gene Expression Array v2 (Agilent Technologies, USA), representing 39,000+ rat genes and transcripts, was used to assess gene expression in the rat PFC on the third day of extinction training. Sample labeling and hybridization were performed according to the Agilent One-Color Microarray-Based Gene Expression Analysis protocol. Briefly, four pools of RNA (each including the RNA of two rats at equal concentrations) from three experimental groups (AC, YC, and YS) were prepared. A starting amount of 2 μg of total RNA was converted to complementary DNA (cDNA) and transcribed into cRNA in the presence of cyanine 3-UTP. Each complementary RNA (cRNA) sample (1.65 μg) was hybridized for 17 h at 65 °C with rotation and then washed to remove nonspecific hybridization.

Microarrays were scanned using the Agilent Microarray Scanner and Feature Extraction software (v 11.0.1.1) (Agilent Technologies, USA). Data normalization and processing were carried out using the GeneSpring GX software, v. 12.1 (Agilent Technologies, USA). The scatterplot method was used to visualize variations (or reproducibility) in gene expression between arrays (Supplementary Material, Fig. S1).

### RT-Quantitative PCR

The cDNA was synthesized by reverse transcription using total RNA (1 μg) and random hexamer primers with the Transcriptor High Fidelity cDNA Synthesis Kit (Roche, USA) following the manufacturer’s protocol.

All PCR reagents were purchased from Life Technologies (USA) if not otherwise indicated. Real-time PCR analysis was performed in duplicate on a 96-well plate using the Bio-Rad CFX96 Touch™ Real-Time PCR Detection System. The 10-μl PCR reaction contained 4.5 μl of cDNA (diluted 1:2 in nuclease-free water), 5 μl of 2× TaqMan Expression Master Mix, and 0.5 μl of TaqMan™ Gene Expression Assays for ten selected genes and one endogenous control (*Hprt1*) (assay ID numbers used are listed in Table 1). PCR cycling conditions were as follows: an initial step of 95 °C for 10 min, followed by 40 cycles of 95 °C for 15 s and then 60 °C for 60 s. The threshold cycle (Ct) was collected using the CFX Manager*™* software. The relative expression of target genes was calculated by comparative Ct method (2^−ΔΔCt^). Our previous analysis showed that the transcript of *Hprt1* gene was more stable than *Gapdh*; therefore, it served as a normalization control in this analysis.

**Table 1:** The ID number of primers/probe used in RT-qPCR

Gene symbol	Assay ID number
*Brd1*	Rn01407551_m1
*Dot1l*	Rn01535507_m1
*Hist1h2ba*	Rn00575310_s1
*Hist1h2bh*	Rn01530118_m1
*Kdm5a*	Rn01459689_m1
*Kdm6a*	Rn01430760_m1
*Kdm6b*	Rn01471506_m1
*Kdm7a*	Rn01500667_m1
*Smarcc1*	Rn01245449_m1
*Hprt1*	Rn01527840_m1

### Total Histone Extraction

Total histones were extracted from the PFC using EpiQuik™ Total Histone Extraction Kit (Epigentek Group Inc., USA) following the manufacturer’s instruction. Briefly, 30–45 mg of frozen tissue was homogenized in the presence of ceramic beads (*ø* = 2.8 mm) and 250 μl of 1× pre-lysis buffer using the Bioprep-24 Homogenizer (Aosheng, China). The sample was then transferred to a new tube and centrifuged at 10,000 rpm for 1 min at 4 °C. The pellet was resuspended in 75 μl of lysis buffer and incubated on ice for 30 min. After centrifugation (12,000 rpm for 5 min at 4 °C), the supernatant fraction was mixed with DTT-containing balance buffer. Protein concentrations were measured with the BCA Protein Assay Macro Kit (Serva, Germany); the extracts were aliquoted and stored at −80 °C.

### Western Blot

For Western blot, 10 μg of protein from each sample was denatured in the presence of 4× sample buffer at 95 °C for 5 min before separation on a 15% SDS-PAGE using the Mini-PROTEAN Tetra System (Bio-Rad, USA). Proteins were transferred to 0.2 μm low-fluorescence PVDF transfer membranes (Thermo Fisher, USA) using the Pierce G2 Fast Blotter (Thermo Scientific, USA). Membranes were blocked with 5% BSA dissolved in Tris-buffered saline containing 0.1% Tween-20 (TBST 0.1%) for 1 h at room temperature. Then, blots were incubated overnight at 4 °C (gentle agitation) with specific primary antibodies (Table 2) diluted in 1% BSA-TBST 0.1%. The incubation with anti-rabbit IgG or anti-mouse IgG, HRP-linked antibodies (1:2000 diluted in 1% BSA-TBST 0.1%, Cell Signaling, USA) was performed at room temperature. Primary and secondary antibody incubation was followed by four washes in TBST 0.1% (6 min, rocking, room temperature).

**Table 2:** The primary antibodies used in Western blot analysis

Antibodies	Cat no.
H3K9ac	#9649
H3K14ac	#7627
H3K18ac	#13998
H3K27ac	#8173
H4K5ac	#8647
H4K8ac	#2594
H4K12ac	#13944
H3K4me2	#9725
H3K4me3	#9751
H3K9me2	#4658
H3K9me3	#13969
H3K27me2	#9728
H3K27me3	#9733
H3K79me2	#5427
H3K79me3	#4909
Histone H3	#4499
Histone H4	#2935

All antibodies with 1:1000 dilution were used and purchased from Cell Signaling, USA

The blots were developed with the enhanced chemiluminescence (ECL) reagent (WesternBright Sirius, Advansta, CA, USA), imaged on an LAS-4000 imaging system (Fujifilm, USA), and quantified using the Multi Gauge software (v. 3.0, Fujifilm). To correctly assess the difference in histone acetylation and methylation marks, the blots were normalized to total histone H3 or H4.

### Statistical Analysis

The data are expressed as the means ± SEM. The behavioral data were analyzed by two-way ANOVA for repeated measures followed by a post hoc Newman-Keuls’ test using the Statistica v.10 software. The statistical analysis of molecular data was conducted using the Prism software (GraphPad Software, v. 7.0). The statistical differences between gene expression levels and Western blot data were assessed by one-way ANOVA with Dunnett’s post hoc test; significance was set at *p* < 0.05.

R software (v. 3.1.2) was used to conduct the unpaired Student’s *t* test and one-way ANOVA followed by the Benjamini and Hochberg false discovery rate (FDR) for multiple comparisons for correction of microarray data. To select differentially expressed genes between the AC and YS groups, cutoffs were set as FDR ≤0.1 and values of log_2_ FC ≥1.0 or ≤−1.0 were used.

## Results

### Behavioral Analysis

During 12–14 self-administration sessions in C-SA, ExT-3, and ExT-10 groups, animals received from 110 to 131 mg/kg of cocaine. The animals showed stable lever pressing during the last three self-administration days, with less than a 10% difference in their daily intake of cocaine. Rats self-administering cocaine in C-SA pressed significantly more frequently on the active than on the inactive lever from the 2nd till 13th day of self-administration, as assessed by the lever × day session interaction (*F*_(12,120)_ = 3.674, *p* < 0.000). Rats self-administering cocaine in ExT-3 and ExT-10 pressed significantly more frequently on the active than on the inactive lever from the 2nd day of self-administration until the 1st day of abstinence, *F*_(14,168)_ = 3.76; *p* < 0.001 and *F*_(20,280)_ = 7.84; *p* < 0.000, respectively. In the yoked cocaine and saline groups, no revealed significant difference in pressing the active vs. the inactive lever was observed.

### Profiling Gene Expression During Extinction Training Followed Cocaine Self-Administration

**Fig. 2:**
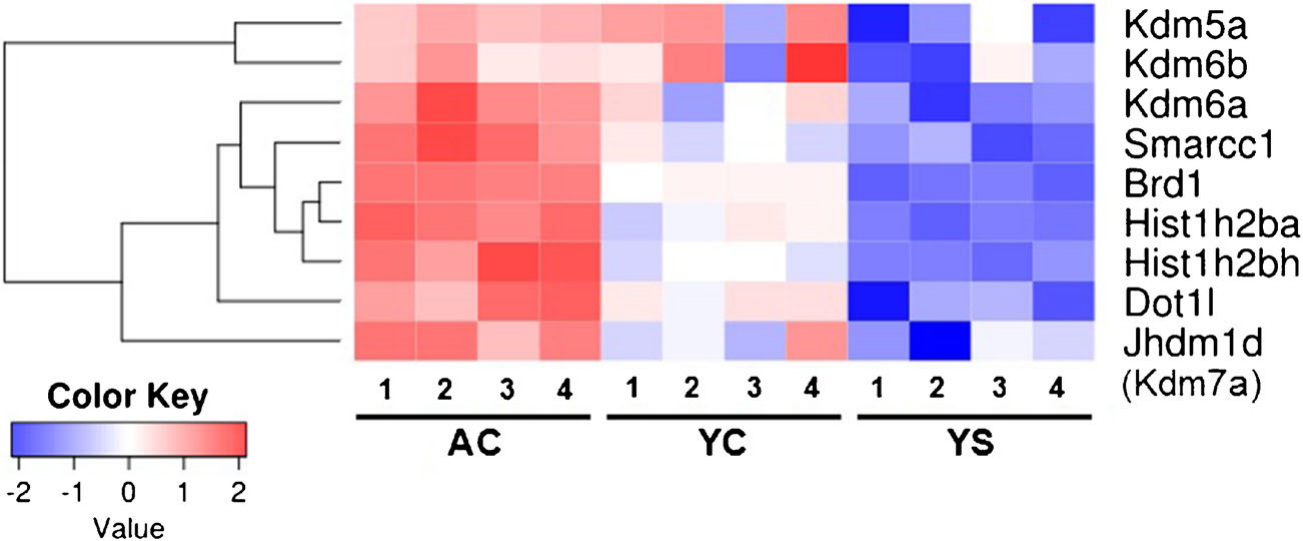
Gene expression changes in the rat PFC on the third extinction training day followed cocaine self-administration. Microarray data from one-way ANOVA are shown as a heat map displaying expression of the selected genes. The *intensity* of the *color* is proportional to the standardized values (between −2.0 and 2.0) from each microarray, as displayed on the *bar below the heat map images*. *AC* active cocaine rats, *YC* yoked cocaine rats, *YS* yoked saline rats (control). The numbers from *1* to *4 below heat maps* indicate the number of pooled samples (*n* = 2/pool)

The influence of extinction training following cocaine self-administration on the transcriptome in the PFC was analyzed using the microarray approach. We found a total of 416 substantially altered transcripts (334 up- and 82 downregulated) in AC rats compared to YS control (Supplementary Material, Table S1). To increase our understanding of the functional role of these genes, we performed Gene Ontology (GO) analysis using the STRING version 10.0 database. With respect to cellular components, some important groups were identified including the axon part, Golgi apparatus, chromatin, synapse part, and neuron projection (Supplementary Material, Table 2S). In terms of molecular functions, three major groups were involved in ion binding, catalytic activity, and transcription factor activity (Supplementary Material, Table 2S). Subsequently, further analysis in three experimental groups of rats (AC, YC, and YS) focused on genes encoding specific types of histones and histone-modifying enzymes involved in PTMs that may play pivotal roles in chromatin remodeling. The heat map showed nine genes involved in chromatin remodeling that were selected for further real-time PCR analysis. The expression levels of 7 out of 9 genes (*Kdm6a*, *Smarcc1*, *Brd1*, *Hist1h2ba*, *Hist1h2bh*, *Dot1l*, and *Kdm7a/Jhdm1d*) were higher in the AC group compared to the YC and YS groups; the transcripts of two genes (*Kdm5a* and *Kdm6a*) seemed to be upregulated in both AC and YC groups (Fig. 2).

### Gene Expression During Cocaine Self-Administration and Extinction Training

**Fig. 3:**
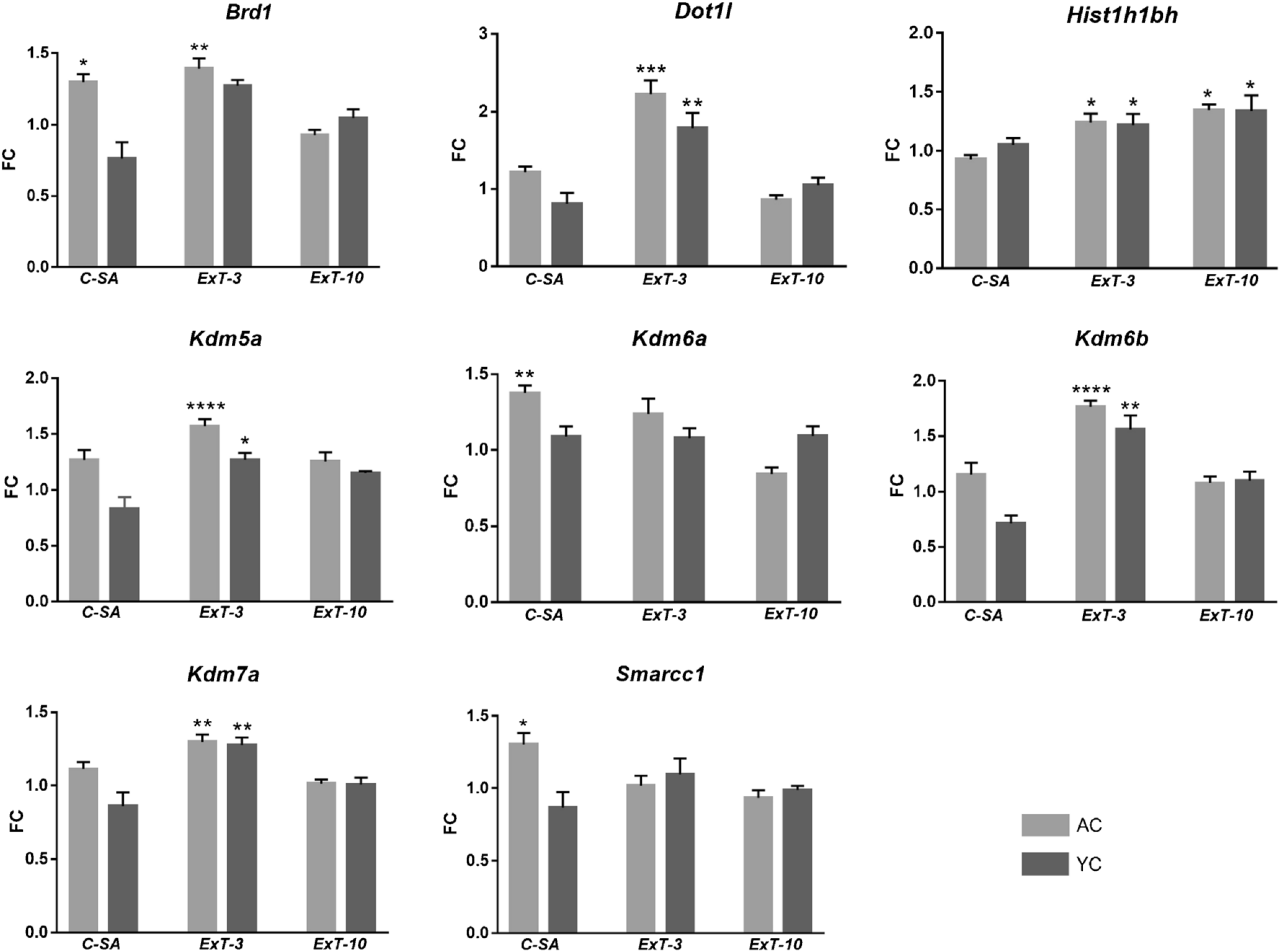
The relative amount of the mRNA transcripts in the rat PFC during cocaine self-administration and cocaine abstinence with extinction training. *C-SA* cocaine self-administration experiment, *ExT-3* cocaine self-administration with 3-day extinction training, *ExT-10* cocaine self-administration with 10-day extinction training, *AC* active cocaine rats, *YC* yoked cocaine rats. Fold change expressed as 2^−ΔΔCt^ was normalized to the mean expression of the control (*YS* yoked saline rats). Significance levels were calculated by one-way ANOVA with post hoc Dunnett’s test (*p ≤ 0.05, **p ≤ 0.01, ***p ≤ 0.001, ****p ≤ 0.0001 vs. YS group; *N* = 6 animals/group; *error bars* ± SEM)

To confirm whether the increases in the expression level of the nine selected genes occurred in three experiments, quantitative real-time PCR was performed. The amplification of the *Hist1h2ba* gene was unsatisfactory so we excluded it from subsequent analysis. The relative levels of messenger RNA (mRNA) transcripts and one-way ANOVA results are presented in Fig. 3 and Table 3. We found that transcripts of *Kdm6a* and *Smarcc2* genes were enriched directly during cocaine self-administration, but the expression of *Brd1* increased during both cocaine self-administration and after the third day of cocaine abstinence with extinction training. Interestingly, we observed that the transcript levels of five genes (*Dot1l*, *Kdm5a*, *Kdm6a*, *Kdm6b*, and *Kdm7a/Jhdm1d)* substantially increased in both cocaine self-administering and cocaine passively receiving rats solely on the third day of extinction. Surprisingly, the level of mRNA *Hist1h2bh* was slightly elevated during both early and late phases of cocaine abstinence in rats receiving cocaine actively and passively.

**Table 3:** The fold change for differentially regulated genes in three experiments assessed by RT-qPCR

Gene symbol	Phase of experiment	Group of rats	FC	One-way ANOVA
*Brd1*	C-AC	AC	1.3	*F*_(2,15)_ = 11.93, *p* = 0.0008
	ExT-3	AC YC	1.4 1.3	*F*_(2,15)_ = 5.70, *p* = 0.0131
*Dot1l*	ExT-3	AC YC	2.2 1.8	*F*_(2,15)_ = 16.17, *p* = 0.0002
*Hist1h2bh*	ExT-3	AC YC	1.2 1.2	*F*_(2,15)_ = 5.14, *p* = 0.0199
	ExT-10	AC YC	1.3 1.3	*F*_(2,15)_ = 4.82, *p* = 0.0242
*Kdm5a*	ExT-3	AC YC	1.6 1.3	*F*_(2,15)_ = 20.40, *p* < 0.0001
*Kdm6a*	C-AC	AC	1.4	*F*_(2,15)_ = 8.70, *p* = 0.0031
*Kdm6b*	ExT-3	AC YC	1.8 1.6	*F*_(2,15)_ = 18.29, *p* < 0.0001
*Kdm7a*	ExT-3	AC YC	1.3 1.3	*F*_(2,15)_ = 11.79, *p* = 0.0008
*Smarcc1*	C-AC	AC	1.3	*F*_(2,15)_ = 7.28, *p* = 0.0062

*C-AC* cocaine self-administration, *ExT-3* third day of extinction training followed by cocaine self-administration, *ExT-10* tenth day of extinction training followed by cocaine self-administration, *AC* active cocaine rats, *YC* yoked cocaine rats, *YS* yoked saline rats (control)

### Histone Acetylation and Methylation During Early Extinction Training

**Fig. 4:**
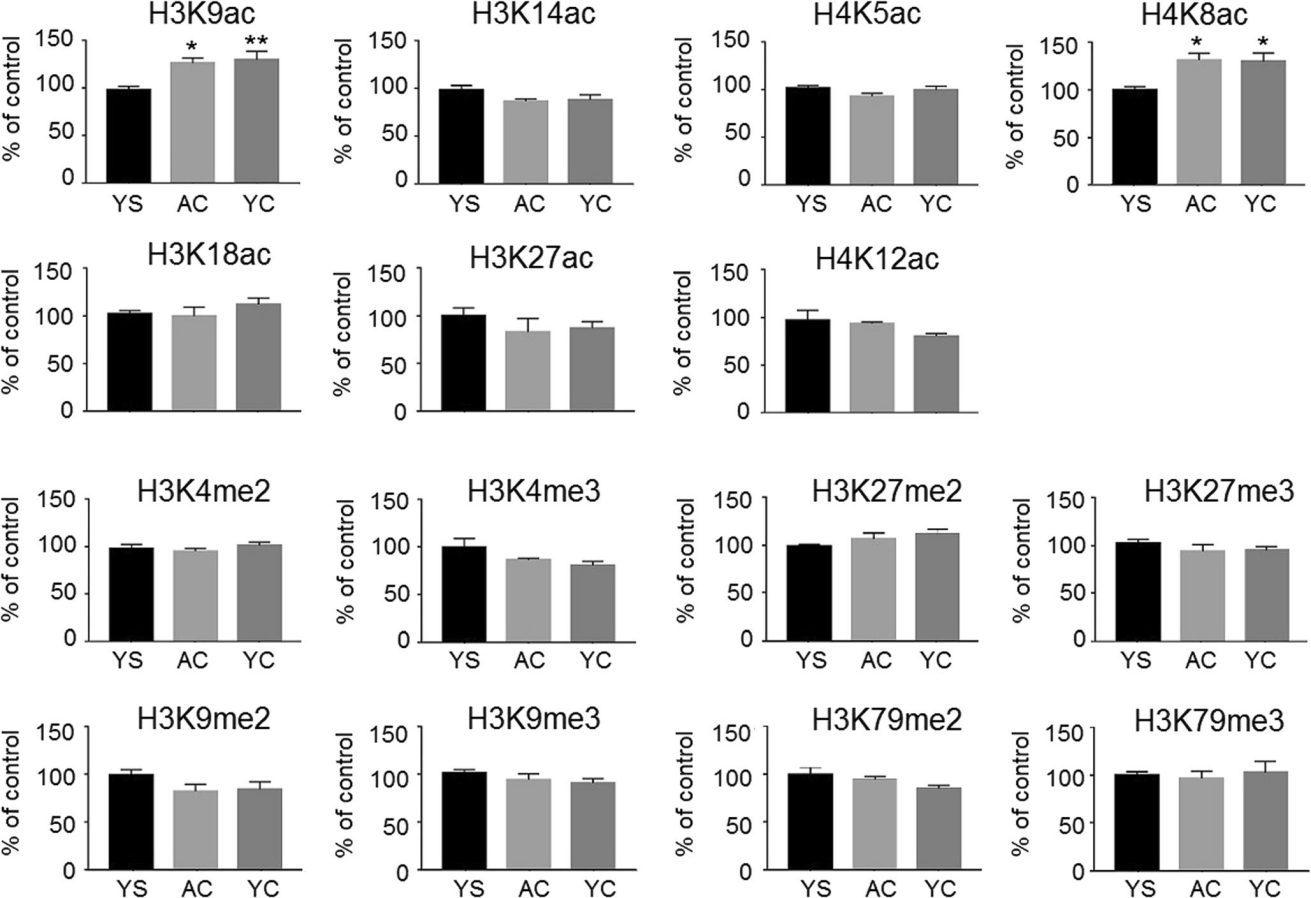
Quantitative profiling of histone acetylation and methylation at specific lysines in the rat PFC on the third day of extinction training following cocaine self-administration. *AC* active cocaine rats, *YC* yoked cocaine rats, *YS* yoked saline rats (control). Significance levels were calculated by one-way ANOVA with post hoc Dunnett’s test (*p ≤ 0.05, **p ≤ 0.01 vs. YS group; *N* = 6 animals/group; *error bars* ± SEM)

In order to determine whether the acetylation and methylation of histone lysines were influenced by changes of the expression level of the genes encoding histone-modifying enzymes, specific antibodies were used. Surprisingly, Western blot analysis showed that the global levels of histone H3 and H4 acetylation and methylation, in most cases, were similar in three experimental groups of rats during early cocaine abstinence (Fig. 4). It is important to emphasize that only acetylation of histone H3 at lysine 9 and histone H4 at lysine 8 increased significantly (*F*_(2,15)_ = 7.685, *p* = 0.0116 and *F*_(2,15)_ = 6.654, *p* = 0.0085, respectively) in rats self-administering cocaine and those receiving yoked cocaine compared to YS control.

## Discussion

In this study, we have shown that cocaine self-administration and/or early period of cocaine abstinence with extinction training induces upregulation of several genes crucial for chromatin remodeling in the rat PFC. The gene overexpression pattern in our experimental approach is associated with (i) cocaine self-administration with *Kdm6a* and *Smarcc1* genes; (ii) cocaine self-administration and early extinction training with *Brd1* gene; (iii) cocaine early withdrawal with *Dot1l*, *Kdm5a*, *Kdm6b*, and *Kdm7a* gene; and (iv) early and late cocaine abstinence with *Hist1h1bh* gene. Interestingly, the majority of analyzed genes encoding histone-modifying enzymes were changed only at early cocaine abstinence with extinction training in rats self-administering cocaine and yoked cocaine controls. Subsequently, our further analysis revealed that the global changes in acetylation and methylation at specific lysine of histones H3 and H4 mostly remained unchanged, however, with the exception of the levels of histone H3 and H4 acetylation (i.e., H3K9ac and H4K8ac) (Fig. 4). Our data have established that the global acetylation of histone H3 at lysine 9 and histone H4 at lysine 8 increased significantly in both AC and YC groups on the third day of extinction training. In contrast, several studies reported that cocaine administration results in the site-specific elevation of H3K9/14ac or H4K5/8/12ac levels in the reward circuitry of rat brain depending on cocaine administration paradigms (Rogge and Wood 2013). Intriguingly, an earlier study revealed that cocaine self-administration followed by 7 days of forced abstinence increased the amount of H3K9/14ac at the *Bdnf IV* promoter in rat medial prefrontal cortex (mPFC) that correlated with its expression (Sadri-Vakili et al. 2010). Moreover, Freeman et al. (2008) showed that cocaine self-administration and forced abstinence for 1 day increased H3K9/14ac at the promoter gene of *Npy*, in rat mPFC, enhancing its transcription.

Most importantly, we demonstrated for the first time an increased level of *Brd1* mRNA after cocaine self-administration and its early abstinence with extinction training in the AC group (Fig. 3). *Brd1* gene encodes a bromodomain-containing protein 1, which is a component of the MOZ/MORF acetyltransferase complex stimulating acetylation of histones H3 and H4 (Fryland et al. 2016). Brd1 is assumed to be a transcriptional regulator (scaffold protein) which belongs to the subfamily IV of Brd proteins but has not been extensively studied for binding to acetylation marks (Ullah et al. 2008; Filippakopoulos and Knapp 2012). However, it was established that Brd1 is important for transcription regulation in the brain by influencing histone acetylation and, thus, the chromatin state (Fryland et al. 2016). In addition, a study by Severinsen et al. (2006) showed that a polymorphism in the *Brd1* gene is associated with schizophrenia and bipolar affective disorder as well as suggesting that Brd1 protein may be involved in neurodevelopment and synaptic plasticity. A growing body of evidence indicates that the regulation of gene expression via chromatin modification and/or remodeling is involved in long-term memory and synaptic plasticity (Malvaez et al. 2010; Barrett and Wood 2008). Interestingly, Malvaez et al. (2010a) confirmed that systemic administration of nonselective HDAC inhibitor, sodium butyrate, that enhanced acetylation of histone H3 in the NAc facilitated the extinction of the cocaine memory and attenuated reinstatement of cocaine-seeking behavior. Since we expect that Brd1 protein has a beneficial effect and may help to maintain gene expression in the PFC by H3K9 and K4K8 hyperacetylation, limiting subsequent cocaine seeking after extinction learning, however, further in vivo studies are needed to fully elucidate whether Brd1 protein indeed is able to attenuate the reinstatement of drug-seeking behavior.

Next, we observed the elevated level of *Dot1l* (more than twofold) in rats after cocaine self-administration and early cocaine abstinence with extinction training only in the AC group (Fig. 3). Dot1l catalyzes histone H3K79 methylation of nucleosomes leading to a concomitant increase in gene expression (Helin and Dhanak 2014). Surprisingly, our Western blot analysis revealed that the levels of H3K79me2 and H3K79me3 were unchanged. To date, there is no literature examining the relationship between *Dot1l* expression and di- and tri-methylation of histone H3 at lysine 79 in a rat model of cocaine self-administration. Furthermore, our data indicated that the transcript levels of *Kdm5a*, *Kdm6b*, and *Kdm7a* increased significantly only on the third day of extinction training in the AC and YC groups. These genes encode proteins that belong to the JmjC domain-containing lysine demethylase family of enzymes that remove methyl groups from lysine residues. The Kdm5a and Kdm6b proteins can catalyze the demethylation of H3K4me2/me3 and H3K27me2/me3, respectively (Dimitrova et al. 2015; Sadakierska-Chudy and Filip 2015), while Kdm7a (also known as Jhdm1d) demethylates histone H3K9 and H3K27 (Horton et al. 2010). Despite the changes in gene expression, we found that the global di- or tri-methylation status of histone H3 at lysine 4, 9, and 27 was similar in all experimental groups of animals. Intriguingly, a recent study has shown that the induction of *Kdm5*, *Kdm6b*, and *Kdm7a* genes in the mouse hippocampal neurons is activity-dependent; however, global changes in H3K27me3 were not observed (Wijayatunge et al. 2014). Our findings of global changes in histone methylation seem to be in line with the latter study, although analysis was performed in different brain structures and under different experimental conditions.

It is worth noting that the expression of *Dot1l*, *Hist1h1bh*, *Kdm5a*, *Kdm6b*, and *Kdm7a* genes was enhanced in rats actively and passively receiving cocaine. Therefore, we do not link this gene upregulation with motivational aspects of cocaine intake, the formation of the new extinction memory accompanied extinction training or depression-like behavior being observed on the third day of cocaine abstinence only in rats withdrawn from cocaine self-administration (Frankowska et al. 2010). We suggest that the changes in gene expression in the PFC are associated with diminished cognitive function, persisting even after abstinence from the drug. In fact, both cocaine-treated groups of animals with self-administration procedure and yoked rats learned Pavlovian associations (Kearns et al. 2005), functions that may be impaired after cocaine experience.

In conclusion, we used a rat model to characterize molecular changes in the PFC during cocaine self-administration and cocaine abstinence with extinction training. We discovered that the transcript levels of genes encoding histone-modifying enzymes promoting the active state of chromatin were significantly elevated mainly in early but not late phase of cocaine abstinence. We postulated that cocaine abstinence with extinction training enhanced the transcriptional activity needed for synaptic plasticity facilitating extinction training. Furthermore, we considered extending the period of the activation state to assess the potential for decreasing drug- and cue-induced drug preference and relapse. Thus, further in vivo functional studies are needed to exclude or confirm our supposition. We strongly believe that observations from this paper can be used in the near future to establish molecular targets related to epigenetics that could facilitate the extinction of cocaine memory and treat drug-seeking behavior.

## Electronic Supplementary Material

ESM 1 (DOC 776 kb)
