# Supplementary material for: Cocaine Administration and Its Withdrawal Enhance the Expression of Genes Encoding Histone-Modifying Enzymes and Histone Acetylation in the Rat Prefrontal Cortex
Source: Neurotox Res. 2017 Apr 10;32(1):141–50. doi: 10.1007/s12640-017-9728-7 (PMC5487868; doi:10.1007/s12640-017-9728-7)
Supplement: Supplementary file 1 — (DOC 776 kb) [file 12640_2017_9728_MOESM1_ESM.doc]

**Neurotoxcity Research**

**Title: Cocaine administration and its withdrawal enhance the expression of genes encoding histone-modifying enzymes and histone acetylation in the rat prefrontal cortex**

**Authors:** Anna Sadakierska-Chudy^1^, Małgorzata Frankowska^1^, Joanna Jastrzębska^1^, Karolina Wydra^1^, Joanna Miszkiel^1^, Marek Sanak^2^, Małgorzata Filip^1^

**Affiliation:**

^1^ Institute of Pharmacology Polish Academy of Sciences, Department of Pharmacology, Laboratory of Drug Addiction Pharmacology, Krakow, Poland

^2^  Jagiellonian University, Medical College, Laboratory of Molecular Biology and Clinical Genetics, Krakow, Poland

**Corresponding author:**

# dr Anna Sadakierska-Chudy

# Institute of Pharmacology Polish Academy of Sciences, Department of Pharmacology, Laboratory of Drug Addiction Pharmacology, ul. Smetna 12, 31-343 Krakow, Poland

# Phone number: +48-12-6623214; Fax number: +48-12-6374500

# e-mail: [**annasc@if-pan.krakow.pl**](mailto:annasc@if-pan.krakow.pl)


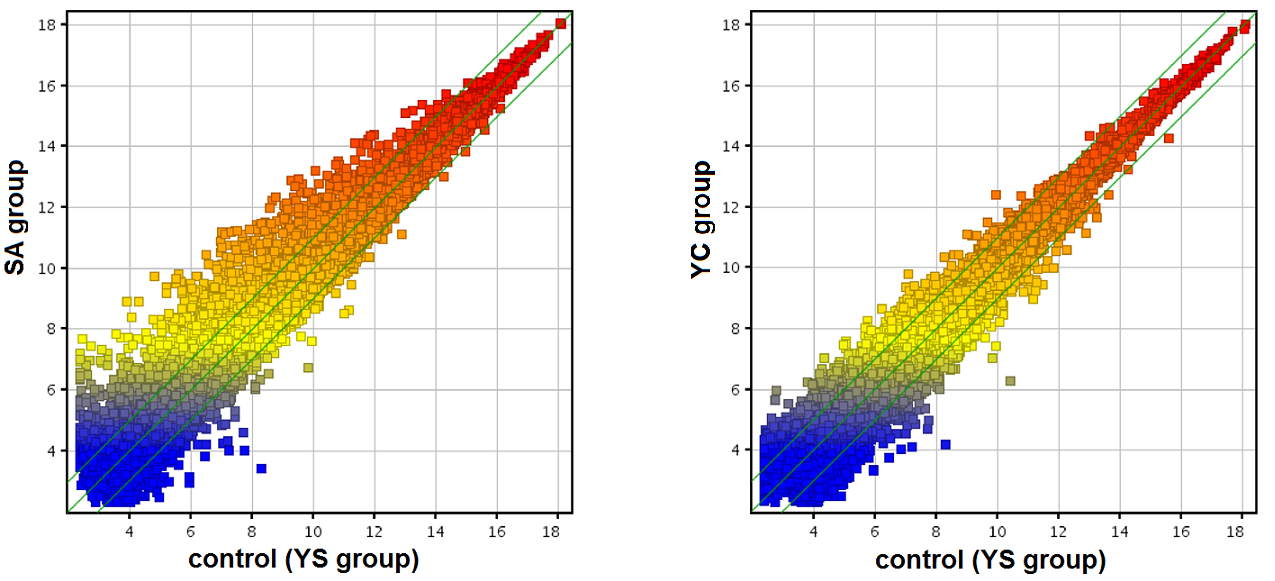


**Fig. S1** Scatter plots showing variation of two data items correlation between signal values of SA and YS groups or YC and YS groups. The values on the X and Y axes are the normalized signal values of the samples (log_2_ scaled) or average normalized signal values of the compared groups (log_2_ scaled). The green lines are Fold Change lines (default fold change value given is 2.0). The genes above the top and below the bottom green lines indicated more than 2.0 fold change of gene expression level between SA and YS or YC and YS groups. Abbreviations: SA – cocaine self-administering rats; YC- yoked cocaine rats; YS – yoked saline rats (control).

**Table S1** Substantially altered transcripts in the rat PFC during early (3. day) cocaine abstinence with extinction training identify by microarray analysis

UPREGULATED GENES

| Gene symbol Probe ID **t-test SA *vs.* YS**  p-value FDR log_2_FC |
| --- |
| | *Aaed1* | A_64_P075421 | 3.32E-05 | 1.85E-03 | 1.10 | | --- | --- | --- | --- | --- | | *Abca5* | A_44_P158609 | 2.00E-04 | 5.97E-03 | 1.07 | | *Abcb1a* | A_44_P279083 | 2.92E-05 | 1.70E-03 | 2.20 | | *Ackr4* | A_64_P027138 | 2.14E-03 | 2.26E-02 | 1.52 | | *Adam7* | A_64_P159300 | 7.26E-03 | 4.48E-02 | 1.21 | | *Adarb1* | A_44_P447650 | 4.44E-03 | 3.36E-02 | 1.06 | | *Aida* | A_44_P130732 | 2.59E-02 | 9.45E-02 | 1.29 | | *Alg13* | A_44_P801714 | 1.35E-04 | 4.78E-03 | 1.58 | | *Amer1* | A_64_P043601 | 5.46E-03 | 3.80E-02 | 1.25 | | *Ankrd34b* | A_64_P025386 | 3.98E-03 | 3.17E-02 | 1.21 | | *Ar* | A_64_P037631 | 1.24E-03 | 1.69E-02 | 2.06 | | *Arcn1* | A_43_P17964 | 1.81E-02 | 7.58E-02 | 1.06 | | *Arhgap25* | A_44_P668582 | 3.97E-06 | 4.60E-04 | 4.32 | | *Arhgap5* | A_44_P990065 | 4.14E-03 | 3.24E-02 | 1.59 | | *Arl6ip6* | A_64_P033792 | 2.03E-06 | 3.03E-04 | 2.68 | | *Armcx1* | A_44_P145891 | 7.41E-06 | 6.88E-04 | 1.86 | | *Arvcf* | A_64_P113621 | 9.08E-07 | 1.87E-04 | 4.51 | | *Asb7* | A_44_P409950 | 7.42E-03 | 4.54E-02 | 1.03 | | *Atad2* | A_44_P385014 | 2.66E-07 | 9.20E-05 | 4.42 | | *Atrx* | A_64_P089125 | 4.56E-03 | 3.41E-02 | 1.80 | | *Axin1* | A_44_P154049 | 1.40E-04 | 4.87E-03 | 1.19 | | *B3gnt2* | A_44_P975327 | 4.73E-03 | 3.49E-02 | 1.43 | | *Bahcc1* | A_64_P041061 | 1.50E-05 | 1.10E-03 | 4.37 | | *Bcl2* | A_64_P005952 | 1.75E-02 | 7.44E-02 | 2.80 | | *Bmp3* | A_64_P101499 | 1.85E-02 | 7.67E-02 | 1.10 | | *Bmx* | A_64_P037736 | 4.17E-03 | 3.25E-02 | 1.04 | | *Brap* | A_64_P077698 | 3.24E-05 | 1.83E-03 | 1.21 | | ***Brd1*** | **A_44_P470043** | **1.13E-07** | **6.24E-05** | **2.66** | | *Bst1* | A_64_P079751 | 1.49E-03 | 1.86E-02 | 1.13 | | *Btla* | A_64_P127803 | 5.90E-04 | 1.13E-02 | 1.13 | | *Cacna1i* | A_64_P017228 | 1.38E-03 | 1.79E-02 | 1.04 | | *Cacul1* | A_44_P222475 | 5.75E-03 | 3.89E-02 | 2.80 | | *Camsap2* | A_44_P970213 | 9.29E-09 | 2.07E-05 | 4.63 | | *Casc4* | A_64_P040125 | 1.12E-02 | 5.75E-02 | 2.22 | | *Cbx3* | A_64_P061602 | 1.30E-02 | 6.30E-02 | 1.91 | | *Ccdc82* | A_44_P943756 | 2.42E-05 | 1.52E-03 | 1.18 | | *Ccnt1* | A_44_P504221 | 8.04E-03 | 4.75E-02 | 1.04 | | *Cd84* | A_44_P698351 | 6.86E-03 | 4.32E-02 | 2.00 | | *Cdca4* | A_44_P962936 | 3.12E-03 | 2.78E-02 | 1.66 | | *Cdk11b* | A_44_P221980 | 4.77E-05 | 2.37E-03 | 2.96 | | *Cers4* | A_64_P105928 | 3.34E-03 | 2.88E-02 | 1.31 | | *Chd2* | A_44_P282794 | 8.02E-03 | 4.75E-02 | 1.04 | | *Chodl* | A_44_P335776 | 1.81E-03 | 2.07E-02 | 1.08 | | *Cnksr2* | A_44_P196440 | 1.62E-03 | 1.94E-02 | 1.44 | | *Cox6c-ps1* | A_64_P058266 | 1.48E-05 | 1.09E-03 | 1.85 | | *Cox7c* | A_44_P540570 | 9.84E-09 | 2.07E-05 | 2.62 | | *Cpg1* | A_64_P027540 | 3.04E-06 | 3.89E-04 | 1.07 | | *Creg2* | A_42_P811632 | 3.90E-03 | 3.13E-02 | 1.01 | | *Crem* | A_44_P988767 | 5.92E-05 | 2.73E-03 | 1.33 | | *Csad* | A_42_P506273 | 3.22E-06 | 4.00E-04 | 2.42 | | *Csgalnact1* | A_64_P144978 | 7.18E-03 | 4.45E-02 | 1.13 | | *Csnk1g3* | A_44_P105004 | 6.77E-03 | 4.29E-02 | 1.25 | | *Csnk2a1* | A_44_P363204 | 2.08E-03 | 2.22E-02 | 1.36 | | *Ctdspl2* | A_64_P047152 | 6.87E-03 | 4.33E-02 | 1.09 | | *Ctps1* | A_64_P095368 | 2.62E-02 | 9.50E-02 | 1.20 | | *Ctsll3* | A_44_P116898 | 7.94E-07 | 1.74E-04 | 2.36 | | *Cwf19l1* | A_64_P020143 | 1.49E-03 | 1.86E-02 | 1.10 | | *Cx3cr1* | A_64_P008406 | 5.75E-03 | 3.89E-02 | 1.41 | | *Cxxc4* | A_64_P080961 | 1.27E-03 | 1.71E-02 | 1.13 | | *Cyp11b2* | A_44_P278418 | 5.67E-05 | 2.65E-03 | 2.08 | | *Cyp2e1* | A_44_P409232 | 6.01E-04 | 1.14E-02 | 1.66 | | *Cys1* | A_44_P792281 | 4.24E-06 | 4.74E-04 | 2.69 | | *Dbr1* | A_64_P047349 | 1.04E-06 | 2.05E-04 | 2.50 | | *Defb51* | A_64_P075562 | 7.87E-05 | 3.31E-03 | 1.40 | | *Dhx16* | A_44_P379461 | 9.75E-10 | 1.51E-05 | 2.70 | | *Dll4* | A_64_P048301 | 1.12E-02 | 5.76E-02 | 2.26 | | *Dlx3* | A_64_P135713 | 3.77E-06 | 4.41E-04 | 1.46 | | *Dmtf1* | A_64_P213481 | 2.47E-02 | 9.17E-02 | 1.52 | | *Dnah9* | A_64_P097083 | 1.13E-02 | 5.79E-02 | 1.22 | | *Dolpp1* | A_43_P15216 | 8.67E-07 | 1.84E-04 | 4.13 | | ***Dot1l*** | **A_64_P023306** | **1.01E-03** | **1.50E-02** | **1.44** | | *Eapp* | A_64_P152311 | 2.74E-07 | 9.31E-05 | 2.65 | | *Efcab1* | A_44_P145060 | 1.07E-02 | 5.61E-02 | 2.20 | | *Efna5* | A_64_P120836 | 8.15E-03 | 4.80E-02 | 2.24 | | *Egr1* | A_44_P233080 | 5.82E-05 | 2.69E-03 | 1.00 | | *Egr2* | A_42_P473594 | 2.21E-03 | 2.30E-02 | 1.48 | | *Eif1ax* | A_64_P076696 | 1.24E-03 | 1.69E-02 | 1.74 | | *Eif4g2* | A_64_P108566 | 1.30E-04 | 4.66E-03 | 1.59 | | *Elac1* | A_64_P160613 | 6.63E-03 | 4.23E-02 | 1.26 | | *Elmo1* | A_44_P592344 | 6.17E-07 | 1.51E-04 | 2.43 | | *Elovl4* | A_44_P558119 | 1.28E-02 | 6.24E-02 | 1.87 | | *Enpp4* | A_44_P272393 | 9.87E-03 | 5.34E-02 | 1.47 | | *Eny2* | A_44_P256181 | 1.46E-02 | 6.76E-02 | 1.70 | | *Epcam* | A_44_P163242 | 1.39E-02 | 6.55E-02 | 1.17 | | *Ergic2* | A_44_P420646 | 1.62E-08 | 2.47E-05 | 3.98 | | *Erlin1* | A_44_P468618 | 3.00E-02 | 1.03E-01 | 1.93 | | *Etaa1* | A_43_P17761 | 1.43E-02 | 6.68E-02 | 1.45 | | *Ets1* | A_64_P097223 | 8.31E-03 | 4.85E-02 | 2.53 | | *Exoc5* | A_44_P188596 | 2.33E-03 | 2.35E-02 | 1.19 | | *F2r* | A_44_P320655 | 3.90E-08 | 3.51E-05 | 4.61 | | *Fam13a* | A_44_P144988 | 4.04E-03 | 3.20E-02 | 1.88 | | *Fank1* | A_44_P518037 | 1.78E-03 | 2.05E-02 | 1.32 | | *Fbxo2* | A_64_P092494 | 1.95E-04 | 5.88E-03 | 1.14 | | *Flt4* | A_64_P140701 | 1.10E-03 | 1.58E-02 | 1.05 | | *Fnbp1l* | A_64_P081987 | 9.10E-03 | 5.11E-02 | 2.15 | | *Fosl1* | A_64_P123118 | 4.87E-03 | 3.55E-02 | 1.54 | | *Foxj3* | A_64_P068648 | 3.67E-03 | 3.03E-02 | 1.58 | | *Frg1* | A_64_P046091 | 8.50E-03 | 4.92E-02 | 1.83 | | *Frs3* | A_64_P058227 | 2.28E-04 | 6.46E-03 | 1.99 | | *Fut11* | A_44_P325534 | 1.51E-02 | 6.87E-02 | 1.40 | | *Gabrb3* | A_44_P306389 | 1.07E-02 | 5.60E-02 | 1.14 | | *Gabrq* | A_64_P105775 | 1.69E-03 | 1.99E-02 | 1.16 | | *Gad1* | A_44_P243102 | 1.46E-02 | 6.74E-02 | 1.26 | | *Gapdh-ps1* | A_64_P005659 | 1.37E-05 | 1.04E-03 | 2.83 | | *Gclm* | A_44_P1011898 | 1.02E-09 | 1.51E-05 | 4.87 | | *Gdap1* | A_43_P20703 | 9.43E-03 | 5.21E-02 | 1.10 | | *Gdpd1* | A_44_P1007438 | 1.08E-03 | 1.56E-02 | 1.03 | | *Ggta1p* | A_64_P023619 | 9.47E-03 | 5.22E-02 | 1.48 | | *Giot1* | A_44_P391035 | 7.88E-04 | 1.32E-02 | 1.26 | | *Gipc2* | A_44_P114500 | 1.73E-05 | 1.20E-03 | 2.14 | | *Gk* | A_64_P123954 | 1.47E-02 | 6.78E-02 | 1.24 | | *Gla* | A_44_P236656 | 3.92E-03 | 3.14E-02 | 1.31 | | *Glcci1* | A_44_P596646 | 4.50E-03 | 3.39E-02 | 1.32 | | *Glt1d1* | A_44_P231655 | 2.33E-02 | 8.85E-02 | 1.83 | | *Gmfb* | A_44_P287089 | 2.83E-03 | 2.65E-02 | 1.12 | | *Gnai1* | A_43_P11734 | 9.88E-03 | 5.34E-02 | 1.53 | | *Gnal* | A_64_P013122 | 1.54E-06 | 2.59E-04 | 1.56 | | *Gnat1* | A_64_P048305 | 4.36E-05 | 2.24E-03 | 2.23 | | *Gpr155* | A_44_P464145 | 5.17E-03 | 3.68E-02 | 1.22 | | *Gpr22* | A_44_P657993 | 2.67E-05 | 1.61E-03 | 1.31 | | *Gpr52* | A_64_P036690 | 7.36E-03 | 4.52E-02 | 1.00 | | *Grid1* | A_64_P082281 | 5.76E-03 | 3.89E-02 | 1.12 | | *Grm7* | A_43_P12487 | 1.30E-02 | 6.30E-02 | 1.03 | | *Gsta2* | A_43_P13967 | 1.97E-05 | 1.32E-03 | 2.39 | | *Gsta5* | A_64_P080566 | 1.76E-04 | 5.53E-03 | 1.27 | | *Gtf2a1* | A_43_P12161 | 3.57E-03 | 2.98E-02 | 1.24 | | *Gtf3c4* | A_64_P094821 | 1.31E-02 | 6.33E-02 | 1.30 | | *Gtse1* | A_44_P1009603 | 6.10E-05 | 2.80E-03 | 2.61 | | *Hcfc1* | A_64_P067310 | 4.21E-04 | 9.31E-03 | 1.02 | | *Hcn4* | A_64_P054626 | 5.11E-03 | 3.65E-02 | 1.06 | | *Heatr3* | A_44_P380038 | 6.25E-03 | 4.09E-02 | 1.95 | | *Heca* | A_44_P398230 | 3.47E-03 | 2.93E-02 | 1.14 | | *Hgf* | A_44_P367525 | 7.96E-04 | 1.32E-02 | 1.05 | | ***Hist1h2ba*** | **A_64_P020303** | **2.85E-07** | **9.37E-05** | **2.49** | | ***Hist1h2bh*** | **A_64_P162197** | **1.02E-05** | **8.59E-04** | **1.20** | | *Hmmr* | A_44_P351118 | 2.00E-04 | 5.97E-03 | 2.40 | | *Hnf1b* | A_64_P104014 | 5.36E-03 | 3.76E-02 | 1.04 | | *Hnrnpa3* | A_64_P047189 | 3.08E-06 | 3.92E-04 | 3.57 | | *Hnrnpll* | A_44_P1052387 | 3.28E-08 | 3.51E-05 | 2.55 | | *Hook1* | A_44_P963558 | 2.75E-02 | 9.78E-02 | 1.39 | | *Hook3* | A_42_P540687 | 6.06E-03 | 4.02E-02 | 1.35 | | *Hpx* | A_44_P1037806 | 1.66E-02 | 7.22E-02 | 1.06 | | *Id3* | A_64_P139586 | 5.19E-06 | 5.37E-04 | 4.93 | | *Ift20* | A_64_P004023 | 7.49E-06 | 6.94E-04 | 1.31 | | *Igfbp7* | A_64_P048187 | 1.28E-06 | 2.33E-04 | 2.84 | | *Ino80* | A_44_P1051068 | 2.89E-02 | 1.01E-01 | 1.01 | | *Irs1* | A_44_P431109 | 2.39E-04 | 6.64E-03 | 1.01 | | *Itch* | A_64_P006408 | 1.04E-02 | 5.52E-02 | 1.32 | | ***Jhdm1d*** | **A_64_P013609** | **5.05E-03** | **3.63E-02** | **1.28** | | *Kcnh2* | A_43_P12992 | 9.99E-05 | 3.93E-03 | 1.47 | | *Kcnip1* | A_44_P499954 | 3.52E-06 | 4.29E-04 | 1.57 | | ***Kdm5a*** | **A_64_P078006** | **7.14E-03** | **4.43E-02** | **1.47** | | ***Kdm6a*** | **A_44_P1024836** | **1.34E-04** | **4.74E-03** | **1.09** | | ***Kdm6b*** | **A_42_P806899** | **1.62E-02** | **7.13E-02** | **1.29** | | *Klf5* | A_44_P526866 | 3.59E-03 | 2.99E-02 | 1.79 | | *Klhl11* | A_44_P438894 | 8.58E-03 | 4.94E-02 | 1.23 | | *Kpna3* | A_44_P238803 | 1.17E-02 | 5.91E-02 | 1.55 | | *Krcc1* | A_44_P296631 | 2.85E-05 | 1.68E-03 | 1.11 | | *Lactb* | A_44_P1044830 | 4.48E-05 | 2.29E-03 | 1.32 | | *Lpar4* | A_44_P902018 | 1.19E-05 | 9.44E-04 | 2.68 | | *Lpgat1* | A_43_P19819 | 9.12E-03 | 5.11E-02 | 1.06 | | *Lrrc8b* | A_44_P451496 | 3.54E-03 | 2.97E-02 | 1.03 | | *Lrrtm2* | A_44_P698466 | 1.39E-02 | 6.56E-02 | 1.12 | | *Lyc2* | A_64_P215398 | 3.72E-06 | 4.40E-04 | 2.00 | | *Mars2* | A_44_P762596 | 8.35E-07 | 1.80E-04 | 2.64 | | *Mbtps2* | A_64_P154278 | 9.67E-03 | 5.28E-02 | 1.37 | | *Mcm4* | A_64_P033939 | 6.80E-04 | 1.22E-02 | 1.56 | | *Me2* | A_64_P036049 | 9.84E-03 | 5.33E-02 | 1.61 | | *Mef2a* | A_44_P435422 | 8.22E-06 | 7.40E-04 | 2.32 | | *Mex3c* | A_43_P20016 | 1.10E-02 | 5.70E-02 | 3.11 | | *Mfsd8* | A_44_P557338 | 5.54E-04 | 1.09E-02 | 1.72 | | *Mgarp* | A_44_P762856 | 4.63E-05 | 2.34E-03 | 1.08 | | *Mitf* | A_64_P056615 | 1.47E-02 | 6.77E-02 | 1.15 | | *Mob4* | A_44_P998060 | 6.83E-06 | 6.49E-04 | 3.62 | | *Mrps15* | A_64_P088925 | 5.60E-03 | 3.84E-02 | 1.57 | | *Nabp1* | A_44_P1001317 | 4.90E-09 | 2.07E-05 | 3.93 | | *Ncam1* | A_64_P111305 | 1.52E-04 | 5.09E-03 | 1.01 | | *Ncoa4* | A_64_P066923 | 1.40E-02 | 6.60E-02 | 1.22 | | *Ndufaf2* | A_43_P22034 | 4.93E-06 | 5.22E-04 | 2.43 | | *Ndufaf4* | A_64_P122646 | 2.65E-02 | 9.57E-02 | 1.34 | | *Ndufaf4* | A_44_P394011 | 1.98E-02 | 7.99E-02 | 1.30 | | *Nedd9* | A_44_P523372 | 2.68E-02 | 9.64E-02 | 1.41 | | *Neurl1* | A_64_P084683 | 1.78E-05 | 1.23E-03 | 1.01 | | *Nfyb* | A_44_P880152 | 7.85E-04 | 1.31E-02 | 1.58 | | *Nmu* | A_64_P399235 | 1.62E-04 | 5.28E-03 | 1.49 | | *Nova1* | A_44_P109584 | 1.47E-02 | 6.77E-02 | 1.72 | | *Nr1d2* | A_44_P395572 | 2.62E-02 | 9.49E-02 | 2.07 | | *Nr3c1* | A_44_P1003794 | 2.26E-05 | 1.45E-03 | 1.09 | | *Nr4a3* | A_43_P12619 | 7.83E-03 | 4.68E-02 | 1.02 | | *Nrp1* | A_42_P768978 | 1.57E-02 | 7.03E-02 | 2.09 | | *Nsmce2* | A_44_P991459 | 1.09E-05 | 8.94E-04 | 2.36 | | *Nuak1* | A_64_P100389 | 4.51E-03 | 3.39E-02 | 1.48 | | *Nudcd1* | A_44_P513764 | 2.21E-03 | 2.30E-02 | 1.15 | | *Nudt9* | A_44_P386799 | 6.10E-06 | 5.96E-04 | 1.08 | | *Numa1* | A_64_P144218 | 4.85E-07 | 1.32E-04 | 1.77 | | *Nupl2* | A_44_P606085 | 1.03E-02 | 5.47E-02 | 1.00 | | *Obox5* | A_64_P025950 | 1.73E-05 | 1.20E-03 | 1.69 | | *Oprl1* | A_43_P12591 | 1.03E-02 | 5.47E-02 | 1.44 | | *Otop3* | A_44_P548864 | 1.01E-03 | 1.50E-02 | 1.34 | | *Ovch2* | A_64_P007123 | 5.13E-03 | 3.66E-02 | 1.46 | | *Oxnad1* | A_44_P603503 | 5.71E-07 | 1.46E-04 | 5.48 | | *P2ry4* | A_64_P069066 | 2.18E-02 | 8.51E-02 | 1.51 | | *Pak2* | A_42_P689755 | 2.70E-02 | 9.69E-02 | 1.36 | | *Pcdh17* | A_44_P684740 | 6.24E-03 | 4.08E-02 | 1.09 | | *Pcdha3* | A_64_P056460 | 4.08E-03 | 3.21E-02 | 1.46 | | *Pcif1* | A_64_P162183 | 4.75E-06 | 5.12E-04 | 1.74 | | *Pde3b* | A_44_P1004645 | 4.88E-05 | 2.39E-03 | 1.08 | | *Pde4b* | A_64_P051631 | 8.69E-03 | 4.97E-02 | 1.48 | | *Pex26* | A_64_P126291 | 5.96E-07 | 1.48E-04 | 3.56 | | *Phb-ps1* | A_64_P157579 | 3.58E-04 | 8.44E-03 | 1.38 | | *Phf14* | A_64_P117914 | 9.67E-03 | 5.28E-02 | 1.05 | | *Phka1* | A_44_P181039 | 1.24E-03 | 1.69E-02 | 1.08 | | *Pigg* | A_44_P704181 | 3.26E-03 | 2.85E-02 | 1.98 | | *Pigm* | A_43_P15300 | 7.84E-03 | 4.68E-02 | 1.04 | | *Pik3ca* | A_64_P111214 | 1.50E-02 | 6.85E-02 | 1.46 | | *Pik3r3* | A_44_P343060 | 1.49E-02 | 6.82E-02 | 1.67 | | *Pin4* | A_64_P088193 | 1.08E-05 | 8.85E-04 | 1.40 | | *Pja2* | A_44_P1009892 | 5.08E-04 | 1.04E-02 | 1.05 | | *Pkia* | A_44_P167951 | 2.48E-02 | 9.18E-02 | 1.34 | | *Pla2g4e* | A_64_P020358 | 2.04E-02 | 8.15E-02 | 1.25 | | *Pmaip1* | A_64_P162029 | 6.13E-03 | 4.05E-02 | 2.11 | | *Pml* | A_64_P090518 | 2.27E-03 | 2.32E-02 | 1.72 | | *Pou5f1* | A_64_P000271 | 1.06E-02 | 5.56E-02 | 1.28 | | *Ppil4* | A_44_P538725 | 8.59E-03 | 4.94E-02 | 1.03 | | *Ppm1b* | A_44_P156450 | 1.18E-02 | 5.92E-02 | 1.44 | | *Ppm1e* | A_44_P313558 | 6.98E-03 | 4.37E-02 | 1.21 | | *Pramef25* | A_64_P055497 | 4.65E-03 | 3.45E-02 | 1.55 | | *Prdm11* | A_64_P165933 | 2.48E-04 | 6.79E-03 | 1.21 | | *Prkaa2* | A_44_P154112 | 8.96E-03 | 5.06E-02 | 1.26 | | *Prkg2* | A_44_P123381 | 4.94E-03 | 3.58E-02 | 1.09 | | *Ptges3l1* | A_44_P269865 | 2.29E-03 | 2.33E-02 | 3.56 | | *Ptgs2* | A_64_P129316 | 1.61E-02 | 7.11E-02 | 2.22 | | *Pwwp2a* | A_44_P259152 | 1.40E-05 | 1.06E-03 | 2.61 | | *Rab11fip3* | A_64_P064493 | 2.02E-06 | 3.02E-04 | 1.26 | | *Rab14* | A_44_P205883 | 2.29E-03 | 2.33E-02 | 1.23 | | *Rab9b* | A_44_P400958 | 2.46E-02 | 9.16E-02 | 2.00 | | *Rad54l2* | A_42_P833106 | 5.66E-03 | 3.86E-02 | 1.01 | | *Rarg* | A_43_P16284 | 4.05E-08 | 3.51E-05 | 3.73 | | *Rasgrp1* | A_43_P11980 | 1.52E-02 | 6.91E-02 | 1.69 | | *Rasl2-9* | A_44_P168184 | 1.15E-05 | 9.23E-04 | 1.17 | | *Rassf5* | A_64_P017298 | 9.36E-03 | 5.19E-02 | 1.30 | | *Rbm27* | A_44_P701356 | 4.70E-03 | 3.47E-02 | 1.69 | | *Rchy1* | A_44_P1025750 | 5.75E-06 | 5.76E-04 | 1.99 | | *Reep5* | A_44_P941342 | 4.42E-04 | 9.60E-03 | 1.07 | | *Ren* | A_64_P059056 | 3.03E-06 | 3.89E-04 | 1.25 | | *Rgcc* | A_64_P150904 | 1.32E-07 | 6.57E-05 | 3.37 | | *Ric8b* | A_44_P198550 | 1.93E-02 | 7.89E-02 | 1.30 | | *Rnf168* | A_44_P403501 | 1.40E-02 | 6.60E-02 | 2.22 | | *Rnmt* | A_44_P1015355 | 1.73E-05 | 1.20E-03 | 1.48 | | *Rrm2b* | A_44_P380274 | 2.24E-03 | 2.31E-02 | 1.85 | | *Rsg1* | A_64_P087355 | 3.18E-03 | 2.81E-02 | 1.18 | | *Rtn4rl2* | A_64_P050463 | 3.31E-05 | 1.85E-03 | 1.31 | | *S100b* | A_43_P15530 | 1.12E-06 | 2.14E-04 | 3.90 | | *Satb2* | A_64_P096277 | 1.25E-02 | 6.15E-02 | 1.16 | | *Scnn1a* | A_43_P12580 | 5.66E-05 | 2.65E-03 | 1.64 | | *Setl1* | A_64_P057143 | 1.84E-04 | 5.66E-03 | 1.65 | | *Sfr1* | A_44_P175167 | 1.27E-02 | 6.23E-02 | 1.16 | | *Sh3rf1* | A_44_P128042 | 6.36E-03 | 4.13E-02 | 2.06 | | *Siglec8* | A_64_P090251 | 2.56E-04 | 6.89E-03 | 1.31 | | *Sik1* | A_64_P147674 | 1.50E-02 | 6.87E-02 | 1.80 | | *Slc16a14* | A_64_P128590 | 2.03E-04 | 6.03E-03 | 1.04 | | *Slc31a1* | A_43_P13226 | 1.95E-04 | 5.88E-03 | 1.80 | | *Slc3a1* | A_64_P074875 | 1.99E-03 | 2.18E-02 | 1.55 | | *Slc41a2* | A_44_P878617 | 6.40E-03 | 4.13E-02 | 1.09 | | *Slc45a4* | A_44_P605002 | 7.20E-06 | 6.78E-04 | 4.97 | | *Slc4a4* | A_64_P016549 | 5.27E-04 | 1.07E-02 | 1.05 | | *Slk* | A_44_P381906 | 7.49E-03 | 4.56E-02 | 1.49 | | ***Smarcc1*** | **A_64_P066631** | **4.88E-05** | **2.39E-03** | **1.04** | | *Smc2* | A_44_P323933 | 2.61E-02 | 9.48E-02 | 1.38 | | *Smoc1* | A_44_P116369 | 4.84E-03 | 3.54E-02 | 1.43 | | *Sms* | A_44_P323914 | 3.95E-03 | 3.16E-02 | 1.15 | | *Snca* | A_64_P160635 | 1.61E-02 | 7.11E-02 | 3.60 | | *Snrnp48* | A_44_P1023480 | 2.83E-04 | 7.30E-03 | 2.16 | | *Snx15* | A_64_P144975 | 4.87E-08 | 3.92E-05 | 5.17 | | *Socs2* | A_42_P543004 | 1.31E-02 | 6.32E-02 | 1.09 | | *Socs6* | A_44_P991940 | 7.41E-06 | 6.88E-04 | 4.31 | | *Sox17* | A_44_P483712 | 2.82E-03 | 2.64E-02 | 1.46 | | *Spata9* | A_64_P112679 | 7.29E-03 | 4.49E-02 | 1.45 | | *Spats2l* | A_44_P364447 | 6.38E-04 | 1.18E-02 | 1.33 | | *Sult1d1* | A_64_P037071 | 1.13E-05 | 9.13E-04 | 3.78 | | *Supt16h* | A_44_P651053 | 8.66E-08 | 5.48E-05 | 3.20 | | *Susd1* | A_44_P497067 | 7.81E-04 | 1.31E-02 | 1.00 | | *Suv39h2* | A_44_P161509 | 5.70E-03 | 3.88E-02 | 1.11 | | *Syngr4* | A_64_P135690 | 6.59E-07 | 1.56E-04 | 2.51 | | *Syt1* | A_44_P149993 | 1.64E-02 | 7.17E-02 | 1.83 | | *Tacc3* | A_64_P055994 | 5.96E-06 | 5.86E-04 | 1.05 | | *Tapt1* | A_64_P098996 | 2.11E-02 | 8.33E-02 | 1.06 | | *Tbca* | A_64_P006226 | 3.75E-06 | 4.41E-04 | 2.81 | | *Tceal5* | A_44_P979417 | 1.91E-04 | 5.80E-03 | 2.69 | | *Tcf7l1* | A_64_P013324 | 4.64E-05 | 2.34E-03 | 1.41 | | *Tdpoz1* | A_64_P120196 | 5.60E-03 | 3.84E-02 | 1.25 | | *Tenm1* | A_64_P015816 | 1.40E-03 | 1.80E-02 | 2.59 | | *Thumpd1* | A_44_P548303 | 1.30E-02 | 6.30E-02 | 1.85 | | *Timm8a2* | A_64_P090375 | 4.79E-03 | 3.51E-02 | 1.17 | | *Tlx3* | A_64_P135643 | 3.03E-02 | 1.04E-01 | 1.02 | | *Tma7* | A_64_P039170 | 6.76E-04 | 1.22E-02 | 1.00 | | *Tmem106a* | A_44_P992056 | 7.50E-04 | 1.28E-02 | 1.19 | | *Tmem132b* | A_64_P075013 | 5.60E-03 | 3.84E-02 | 1.44 | | *Tmem43* | A_64_P149511 | 3.08E-07 | 9.82E-05 | 1.89 | | *Tmem47* | A_64_P030140 | 3.75E-05 | 2.01E-03 | 1.47 | | *Tmem64* | A_44_P138105 | 7.72E-03 | 4.65E-02 | 1.18 | | *Tmf1* | A_44_P444247 | 2.22E-03 | 2.30E-02 | 1.61 | | *Tnfaip8l3* | A_64_P050665 | 1.54E-03 | 1.89E-02 | 1.09 | | *Tob1* | A_44_P212552 | 6.55E-06 | 6.30E-04 | 1.90 | | *Tp53bp1* | A_64_P004575 | 1.78E-02 | 7.51E-02 | 1.24 | | *Tpsg1* | A_44_P383949 | 3.43E-03 | 2.91E-02 | 1.36 | | *Tpt1* | A_44_P398792 | 4.11E-06 | 4.66E-04 | 1.60 | | *Trim33* | A_44_P110035 | 2.59E-02 | 9.45E-02 | 1.14 | | *Trio* | A_44_P247615 | 4.10E-06 | 4.66E-04 | 1.67 | | *Trip12* | A_64_P020571 | 3.80E-04 | 8.77E-03 | 1.18 | | *Tsfm* | A_64_P072442 | 2.14E-05 | 1.39E-03 | 1.91 | | *Tsg101* | A_44_P358110 | 1.08E-06 | 2.08E-04 | 1.88 | | *Ttc28* | A_44_P170018 | 4.42E-05 | 2.27E-03 | 1.59 | | *Tulp1* | A_44_P133366 | 1.57E-04 | 5.19E-03 | 1.42 | | *Uap1* | A_64_P055323 | 2.53E-06 | 3.51E-04 | 2.34 | | *Ubxn2a* | A_64_P039896 | 5.29E-03 | 3.73E-02 | 1.37 | | *Uxs1* | A_64_P092728 | 2.17E-02 | 8.48E-02 | 1.67 | | *Vkorc1l1* | A_44_P1037275 | 1.13E-03 | 1.61E-02 | 1.02 | | *Vps13a* | A_64_P163971 | 4.05E-03 | 3.20E-02 | 2.35 | | *Wbscr17* | A_44_P550559 | 2.85E-03 | 2.65E-02 | 1.36 | | *Wdr82* | A_64_P087341 | 1.34E-03 | 1.76E-02 | 1.53 | | *Whamm* | A_44_P1059322 | 5.03E-07 | 1.36E-04 | 2.44 | | *Xkr4* | A_64_P104713 | 1.94E-02 | 7.90E-02 | 1.03 | | *Xpot* | A_64_P262041 | 1.20E-02 | 6.01E-02 | 1.25 | | *Xrcc2* | A_44_P652180 | 1.12E-03 | 1.60E-02 | 1.13 | | *Xrn1* | A_44_P1002440 | 3.66E-06 | 4.37E-04 | 2.04 | | *Yap1* | A_64_P302952 | 8.22E-05 | 3.40E-03 | 1.10 | | *Zbtb20* | A_44_P721823 | 2.56E-06 | 3.53E-04 | 1.77 | | *Zfp36* | A_44_P435596 | 9.51E-06 | 8.20E-04 | 2.80 | | *Zfp46* | A_64_P162331 | 2.71E-03 | 2.57E-02 | 1.13 | | *Zfp978* | A_64_P037938 | 3.47E-05 | 1.90E-03 | 1.49 | | *Zfyve28* | A_64_P039996 | 1.30E-06 | 2.33E-04 | 2.08 | | *Zmynd11* | A_44_P400223 | 1.41E-02 | 6.62E-02 | 1.50 | | *Zpbp* | A_44_P682959 | 5.73E-03 | 3.89E-02 | 1.08 | |

**in bold** genes selected for real-time PCR validation

DOWNREGULATED GENES

| Gene symbol Probe ID **t-test SA vs. YS**  p-value FDR log_2_FC |
| --- |
| | *Acap1* | A_64_P100879 | 2.26E-02 | 8.67E-02 | -1.10 | | --- | --- | --- | --- | --- | | *Ak7* | A_42_P795467 | 2.02E-03 | 2.19E-02 | -1.16 | | *Antxr1* | A_64_P127397 | 3.69E-03 | 3.03E-02 | -1.07 | | *Aqp1* | A_44_P299247 | 1.70E-03 | 2.00E-02 | -1.32 | | *Asb15* | A_64_P094230 | 2.57E-02 | 9.40E-02 | -1.04 | | *Atp12a* | A_42_P684885 | 1.20E-02 | 6.00E-02 | -1.10 | | *Bcl2l15* | A_44_P506299 | 7.92E-04 | 1.32E-02 | -1.00 | | *Bpifa2* | A_43_P12746 | 8.54E-03 | 4.92E-02 | -1.10 | | *Card14* | A_64_P094249 | 1.93E-04 | 5.84E-03 | -1.01 | | *Ccdc69* | A_64_P062230 | 9.25E-03 | 5.15E-02 | -1.21 | | *Ccdc73* | A_64_P009624 | 3.90E-04 | 8.93E-03 | -1.04 | | *Ccl7* | A_44_P1022002 | 5.73E-03 | 3.89E-02 | -1.08 | | *Cfhr2* | A_64_P000839 | 7.74E-04 | 1.30E-02 | -1.03 | | *Clic6* | A_44_P449623 | 7.62E-03 | 4.60E-02 | -1.14 | | *Cln3* | A_64_P048868 | 5.89E-04 | 1.13E-02 | -1.10 | | *Cyp1a2* | A_42_P711139 | 1.86E-02 | 7.71E-02 | -1.43 | | *Cyp2b15* | A_64_P021928 | 3.48E-04 | 8.28E-03 | -1.03 | | *Defb10* | A_64_P071993 | 1.76E-04 | 5.53E-03 | -1.09 | | *Defb4* | A_42_P767002 | 1.97E-03 | 2.17E-02 | -1.11 | | *Dlk1* | A_42_P754888 | 1.14E-02 | 5.81E-02 | -1.18 | | *Eno3* | A_64_P054803 | 1.91E-02 | 7.81E-02 | -1.11 | | *Fam48b1* | A_64_P013968 | 4.59E-04 | 9.80E-03 | -1.21 | | *Fat2* | A_42_P550241 | 3.09E-02 | 1.05E-01 | -1.14 | | *Figla* | A_44_P806474 | 1.03E-04 | 4.00E-03 | -2.29 | | *Fmo6* | A_64_P080549 | 7.11E-05 | 3.10E-03 | -1.18 | | *Fscb* | A_44_P606240 | 6.56E-04 | 1.20E-02 | -1.12 | | *Galp* | A_64_P162115 | 2.96E-03 | 2.71E-02 | -1.31 | | *Galr1* | A_43_P11633 | 2.89E-02 | 1.01E-01 | -1.17 | | *Gata5* | A_42_P502440 | 4.81E-04 | 1.01E-02 | -1.45 | | *Gbx2* | A_64_P157066 | 3.88E-04 | 8.90E-03 | -1.21 | | *Gcgr* | A_42_P621872 | 2.19E-02 | 8.51E-02 | -1.36 | | *Hes3* | A_64_P148378 | 3.49E-03 | 2.94E-02 | -1.08 | | *Hoxb7* | A_64_P080434 | 1.17E-02 | 5.89E-02 | -1.37 | | *Igfbp6* | A_44_P378799 | 9.20E-04 | 1.43E-02 | -1.09 | | *Il12rb2* | A_64_P007238 | 6.95E-03 | 4.36E-02 | -1.43 | | *Il17c* | A_64_P042995 | 6.42E-03 | 4.14E-02 | -1.03 | | *Il18rap* | A_44_P397803 | 5.22E-03 | 3.70E-02 | -1.10 | | *Impg1* | A_64_P112111 | 5.33E-04 | 1.08E-02 | -1.04 | | *Irx5* | A_64_P094050 | 3.88E-03 | 3.12E-02 | -1.15 | | *Katnal2* | A_64_P027255 | 1.70E-02 | 7.30E-02 | -1.17 | | *Kcnmb3* | A_44_P330736 | 8.24E-05 | 3.40E-03 | -1.02 | | *Klhl10* | A_44_P463613 | 3.45E-03 | 2.92E-02 | -1.18 | | *Krtap4-3* | A_64_P000799 | 1.93E-02 | 7.87E-02 | -1.34 | | *Lepre1* | A_64_P059217 | 2.40E-03 | 2.40E-02 | -1.07 | | *Lgals2* | A_42_P743682 | 7.79E-03 | 4.66E-02 | -1.04 | | *Lgals5* | A_44_P423691 | 4.07E-04 | 9.09E-03 | -1.14 | | *Lipg* | A_64_P151151 | 1.67E-02 | 7.25E-02 | -1.20 | | *Ltbp2* | A_64_P124473 | 4.47E-03 | 3.38E-02 | -1.02 | | *Ly49i9* | A_64_P126896 | 1.07E-02 | 5.60E-02 | -1.25 | | *Lzts2* | A_64_P077021 | 1.05E-02 | 5.55E-02 | -1.12 | | *Mcpt1l4* | A_64_P105988 | 1.12E-02 | 5.74E-02 | -1.23 | | *Mmp13* | A_42_P606126 | 2.09E-02 | 8.27E-02 | -1.28 | | *Mpz* | A_64_P036970 | 2.20E-02 | 8.53E-02 | -1.86 | | *Ms4a8* | A_64_P035808 | 2.68E-03 | 2.56E-02 | -1.13 | | *Myo3a* | A_64_P097183 | 2.14E-03 | 2.26E-02 | -1.24 | | *Myo5c* | A_42_P803810 | 6.16E-03 | 4.06E-02 | -1.01 | | *Myoc* | A_42_P547246 | 5.75E-04 | 1.11E-02 | -1.05 | | *Napsa* | A_42_P738549 | 1.05E-02 | 5.55E-02 | -1.36 | | *Nfatc3* | A_64_P077002 | 1.20E-02 | 6.01E-02 | -1.18 | | *Np4* | A_64_P032504 | 1.71E-02 | 7.32E-02 | -1.77 | | *Nrap* | A_64_P026191 | 7.69E-03 | 4.63E-02 | -1.32 | | *Plekhg1* | A_64_P108354 | 2.00E-04 | 5.97E-03 | -1.01 | | *Pou6f2* | A_64_P023744 | 7.05E-04 | 1.24E-02 | -1.20 | | *Prl8a9* | A_44_P395625 | 1.57E-03 | 1.91E-02 | -1.62 | | *Prpmp5* | A_64_P075532 | 3.79E-04 | 8.76E-03 | -1.00 | | *Prss32* | A_64_P155315 | 1.22E-02 | 6.06E-02 | -1.28 | | *Ptpn22* | A_64_P029476 | 2.82E-04 | 7.29E-03 | -1.19 | | *Slc10a1* | A_42_P477732 | 4.03E-04 | 9.05E-03 | -1.07 | | *Slc17a3* | A_42_P555365 | 9.39E-03 | 5.20E-02 | -1.26 | | *Slc36a3* | A_44_P222936 | 3.62E-03 | 3.00E-02 | -1.05 | | *Spic* | A_44_P121902 | 1.14E-03 | 1.62E-02 | -1.37 | | *Spink14* | A_64_P047470 | 5.21E-04 | 1.06E-02 | -1.00 | | *Suv39h1* | A_64_P116981 | 1.50E-02 | 6.85E-02 | -1.03 | | *T2* | A_64_P060614 | 1.06E-03 | 1.55E-02 | -1.17 | | *Tgm2* | A_42_P824489 | 1.50E-04 | 5.07E-03 | -1.42 | | *Tmem247* | A_42_P516860 | 1.35E-04 | 4.78E-03 | -1.02 | | *Tmod4* | A_64_P102545 | 1.51E-02 | 6.87E-02 | -1.28 | | *Tnni1* | A_42_P577677 | 5.70E-03 | 3.87E-02 | -1.41 | | *Tnp1* | A_64_P114099 | 9.57E-04 | 1.45E-02 | -1.13 | | *Trpm6* | A_64_P106021 | 6.34E-04 | 1.18E-02 | -1.09 | | *Tsks* | A_64_P055002 | 2.57E-04 | 6.90E-03 | -1.11 | | *Ttll2* | A_44_P177857 | 3.25E-03 | 2.84E-02 | -1.19 | |

**Table S2** Gene ontology (GO) cellular components and molecular functions terms enriched among differentially expressed genes in SA *vs*. YS group in the rats PFC during early (3. day) cocaine abstinence with extinction training identified using STRING database.

| **CELLULAR COMPONENTS** | | | | | |
| --- | --- | --- | --- | --- | --- |
| **GO** | **Term** | **Number of genes** | **Gene symbol** | **p-value** | **FDR** |
| GO:0033267 | axon part | 8 | *Aqp1*, *Gabrb3*, Grm7, *Hcn4*, *Myoc*, *Nmu*, *Snca*, *Syt1* | 1.77E-02 | 5.13E-01 |
| GO:0005794 | Golgi apparatus | 18 | *Abca5*, *Arcn1*, *Cnksr2*, *Cyp2e1*, *Fut11*, *Ggta1p*, *Grm7*, *Igfbp6*, *Lipg*, *Mmp13*, *Mob4*, *Myoc*, *Pja2*, *Rasgrp1*, *Sh3rf1*, *Snca*, *Tmem43*, *Uxs1* | 1.88E-02 | 5.21E-01 |
| GO:0000785 | chromatin | 6 | *Ar*, *Atrx*, *Hist1h2ba*, *Rasl2-9*, *Sfr1*, *Tnp1* | 2.44E-02 | 6.01E-01 |
| GO:0044456 | synapse part | 14 | *Axin1*, *F2r*, *Fosl1*, *Gabrb3*, *Gad1*, *Grid1*, *Grm7*, *Hcn4*, *Lrrtm2*, *Mob4*, *Nmu*, *Pja2*, *Snca*, *Syt1* | 3.49E-02 | 7.76E-01 |
| GO:0043005 | neuron projection | 20 | *Aqp1*, *Ar*, *Cnksr2*, *Cx3cr1*, *Cyp11b2*, *Fosl1*, *Gabrb3*, *Grid1*, *Grm7*, *Hcn4*, *Kcnip1*, *Mob4*, *Myoc*, *Ncam1*, *Nmu*, *Nrp1*, *Oprl1*, *Ptgs2*, *Snca*, *Syt1* | 4.08E-02 | 8.13E-01 |
| **MOLECULAR FUNCTIONS** | | | | | |
| GO:0043167 | ion binding | 67 | *Abca5*, *Adam7*, *Adarb1*, *Antxr1*, *Ar****,*** *Atp12a*, *Atrx*, *Ccl7*, *Cdk11b*, *Csad*, *Csnk1g3*, *Csnk2a1*, *Cxxc4*, *Cyp11b2*, *Cyp1a2*, *Cyp2b15*, *Egr1*, *Egr2*, *Eno3*, *Fat2*, *Flt4*, *Gad1*, *Gdpd1*, *Ggta1p*, *Gk*, *Gnal*, *Grm7*, *Gsta2*, *Gsta3*, *Hcn4*, *Hpx*, *Kcnip1*, *Lepre1*, *Lipg*, *Ltbp2*, *Mmp13*, *Mob4*, *Ncam1*, *Nr1d2*, *Nr4a3*, *Nrp1*, *Nsmce2*, *P2ry4*, *Pak2*, *Pde3b*, *Pde4b*, *Pja2*, *Ppm1b*, *Ppm1e*, *Prkaa2*, *Prkg2*, *Ptgs2*, *Rab14*, *Rasgrp1*, *Rasl2-9*, *Rassf5*, *Rnf168*, *S100b*, *Sh3rf1*, *Sik1*, *Slc3a1*, *Slk*, *Snx15*, *Syt1*, *Tnni1 Uxs1*, *Zfp36* | 2.25E-04 | 1.82E-01 |
| GO:0003824 | catalytic activity | 62 | *Abca5*, *Adam7*, *Adarb1*, *Alg13*, *Atrx*, *Bst1*, *Cdk11b*, *Cox7c*, *Csad*, *Csnk1g3*, *Csnk2a1*, *Ctdspl2*, *Cyp11b2*, *Cyp2b15*, *Cyp2e1*, *Egr2*, *Eno3*, *Flt4*, *Fut11*, *Gad1*, *Gclm*, *Gdpd1*, *Ggta1p*, *Gk*, *Gnal*, *Gsta2*, *Gsta3*, *Hgf*, *Kcnh2*, *Lepre1*, *Lipg*, *Lyc2*, *Mmp13*, *Nrp1*, *Nsmce2*, *Nudt9*, *Pak2*, *Pde3b*, *Pde4b*, *Phka1*, *Pigm*, *Pja2*, *Ppm1b*, *Ppm1e*, *Prkaa2*, *Prkg2*, *Ptgs2*, *Rab14*, *Rasl2-9*, *Ren*, *Rnf168*, *Rnmt*, *Sh3rf1*, *Sik1*, *Slc3a1*, *Slk*, *Snca Spink14*, *Sult1d1*, *Trip12*, *Uxs1*, *Vkorc1l1* | 9.58E-04 | 4.03E-01 |
| GO:0003700 | transcription factor activity, sequence-specific DNA binding | 12 | *Ar*, *Crem*, *Egr1*, *Ets1*, *Fosl1*, *Hoxb7*, *Id3*, *Mef2a*, *Mitf*, *Nfyb*, *Nr1d2*, *Nr4a3* | 1.88E-03 | 5.70E-01 |
